# American Dental Association

**Published:** 1889-10

**Authors:** 


### AMERICAN DENTAL ASSOCIATION.

THE past meeting of the Association at Saratoga was not up to former sessions held at that place. The attendance was much smaller and the interest in the proceedings was much less evidenced than at former meetings. Few papers were presented and the discussions were very limited, so much so that the editor of the *Cosmos* pronounced the meeting "the least valuable of any held for several years." He further said, "the sections had not made preparation at all adequate to that which had been accomplished for previous sessions, and, with one exception, and a single paper in another section, there was little to indicate that due effort had been made for a creditable presentation of the subjects embraced in the several departments assigned to the respective sections."

This would seem to be throwing the blame of the failure of the meeting upon the chairmen and secretaries of the several sections, which is not just. We had the honor to fill the position of secretary for sections four and seven, and can answer for those two sections that the chairmen and ourselves did our utmost to present a good programme. In our official capacity we wrote personal letters to each and every member of both sections, with only one response, and then, at very considerable inconvenience to ourselves, volunteered to present a lantern exhibit which was to be accredited to both sections. No; the fault did not lie with the officers, they did their duty. The facts in the case are that the profession is largely written out. As we said editorially in the August number of the Journal when opposing the calling of an International Dental Congress, so say we again, "Those who are directly connected with the American Dental Association realize the fact that it is growing harder every year to get suitable material to make a good programme; and why? Simply because of the division of interest on account of the many anniversary meetings of large proportions which have been held in America the past few years." The American Dental Association is in its decadence, and unless something is done to revive interest in this time-honored institution it will surely pass into oblivion. It is therefore proposed to take advantage of the event of the World's Fair to be held in the United States in 1892, and have a memorial meeting at the place where and the time when that celebration shall be held. Such a movement will give the Association a decided impetus forward and tend to endow it with a new lease of life. We have been in correspondence with several prominent members of the Association and all express themselves as highly pleased with the idea. It is none too early to begin to agitate the question, as the matter must be fully canvassed before the next meeting of the Association is held in order to formulate some concerted plan of action. Let us hear from prominent members of the profession in regard to the proposition.

